# Raman micro-spectroscopy as a viable tool to monitor and estimate the ionic transport in epithelial cells

**DOI:** 10.1038/s41598-017-03595-y

**Published:** 2017-06-13

**Authors:** Leonardo Puppulin, Giuseppe Pezzotti, Hongxin Sun, Shigekuni Hosogi, Takashi Nakahari, Toshio Inui, Yasuaki Kumamoto, Hideo Tanaka, Yoshinori Marunaka

**Affiliations:** 1 Department of Molecular Cell Physiology, Kyoto Prefectural University of Medicine Graduate School of Medical Science, Kyoto, 602-8566 Japan; 2 Ceramic Physics Laboratory, Kyoto Institute of Technology, Kyoto, 606-8585 Japan; 3 Department of Bio-Ionomics, Kyoto Prefectural University of Medicine Graduate School of Medical Science, Kyoto, 602-8566 Japan; 4 Saisei Mirai Clinics, Moriguchi, 570-0012 Japan; 5 Department of Pathology and Cell Regulation, Kyoto Prefectural University of Medicine Graduate School of Medical Science, Kyoto, 602-8566 Japan

## Abstract

The typical response to the lowering of plasma Na^+^ concentration and blood pressure in our body involves the release of aldosterone from the adrenal glands, which triggers the reabsorption of sodium in the kidney. Although the effects of aldosterone on this physiological mechanism were extensively studied in the past decades, there are still some aspects to be fully elucidated. In the present study, we propose for the first time a new approach based on Raman spectroscopy to monitor the ionic activity in aldosterone-treated A6 renal epithelial cells. This spectroscopic technique is capable of probing the cells through their thickness in a non-destructive and nimble way. The spectroscopic variations of the Raman bands associated to the O-H stretching of water were correlated to the variations of ionic concentration in the intracellular and extracellular fluids. The increase of Na^+^ concentration gradients was clearly visualized in the cytosol of aldosterone-treated cells. The enhancement of the Na^+^ current density induced by aldosterone was estimated from the variation of the ionic chemical potential across the intracellular space. In addition, the variation of the O-H Raman bands of water was used to quantify the cell thickness, which was not affected by aldosterone.

## Introduction

The daily excretion/reabsorption of electrolytes in the kidney represents a pivotal physiological mechanism to maintain the appropriate fluid balance in our body, which in turn regulates blood pressure and enables acid-base homeostasis. As far as the lowering of the plasma sodium concentration and/or the fall of blood pressure are concerned, the typical body response to these deficiencies involves the release of angiotensin II-stimulated aldosterone from the adrenal glands, which is a mineralocorticoid hormone regulating the reabsorption of sodium ions in the distal tubules of the nephron and the collecting ducts^1–4^. The main action of aldosterone in regulating Na^+^ reabsorption is represented by the stimulated synthesis of two proteins: epithelial sodium channels (ENaC) and serine/threonine-protein kinase (SGK1), via mineralocorticoid receptor^4, 5^. The synthesis of ENaC increases the total concentration of this protein in the apical membrane of the cell, which clearly results in the enhancement of Na^+^ permeability. Differently, the production of SGK1 indirectly affects the number of ENaCs on the apical membrane by phosphorylating and, hence, neutralizing Nedd 4–2, which is responsible for endocytosis of ENaC from the apical membrane to the cytosolic space^6–9^. Although aldosterone has been identified and synthesized since the mid 1950s^10, 11^, only in the past few decades the metabolism and function of this hormone in stimulating epithelial ions exchange have been elucidated, especially as a result of experiments obtained using renin-angiotensin-aldosterone system blockers, such as direct renin inhibitors, angiotensin-converting-enzyme (ACE) inhibitors, type I angiotensin II (AT_1_)-receptor blockers (ARBs) and mineralocorticoid-receptor antagonist (MRAs)^12–15^. Nonetheless, the current knowledge on the ionic transport mechanisms affected by aldosterone still has to be extended to clarify some puzzling aspects, such as, the intracellular alteration of the ionic concentration, the variation of cell volume and the different effects of this hormone on hypovolaemia (sodium retention and potassium conservation) and hyperkalaemia (potassium secretion), also known as aldosterone paradox^12^. Information on transepithelial ion movements are usually achieved by measurements of short-circuit currents in the Ussing chamber^16^ and ENaC activity is studied by patch clamp techniques^17^, but these methods are not capable of measuring the local variation of ionic concentrations in the cytosol. In previous studies, we exploited methods based on the use of an ENaC blocker, amiloride, and short-circuit current measurement technique to determine the transcellular Na^+^ absorption in the epithelial cells^18^. In addition, short-circuit current measurements supported by mathematical simulation enabled to calculate ENaC translocation in the intracellular space^19, 20^. The outcomes of these studies emphasized the necessity of combining different experimental and theoretical approaches to elaborate a synergetic method of analysis aimed at obtaining the most complete picture of the phenomenon under investigation. In the present study, we propose for the first time a new approach based on Raman micro-spectroscopy to monitor ionic activity in epithelial cells treated with aldosterone. This spectroscopic technique is non-destructive and it offers the advantage of micrometric spatial resolution, which is suitable to probe the cells through their thickness in an accurate and nimble way. This analysis was built upon preliminary calibrations of physiological solutions containing different ions and at different concentrations. The spectroscopic variations of the Raman bands associated to the O-H stretching of water were deeply investigated and correlated to the variations of concentration in physiologically relevant solutions, namely fluids in the intracellular and extracellular compartments.

## Methods

### Ionic water solutions

The effect of different ions on the O-H stretching vibration of water was studied preparing series of water solutions containing different ionic species. In addition, we analyzed protein solutions containing bovine serum albumin (lyophilized powder, ≥98%, Sigma-Aldrich) diluted at different concentrations. Table 1 reports the details of the solutions under investigation: each series consisted of solutions with the same kind of solute, but at different concentrations. The protocol of analysis was designed in order to study the influence of each specific couple anion/cation on the vibrational Raman spectrum of H_2_O and, successively, to verify the additivity of their contributions in multi-ion solutions.

**Table 1:** Details of the ionic solutions analyzed by Raman micro-spectroscopy.

Series	1	2	3	4	5	6	7	8
Solute	NaCl	KCl	MgCl_2_	CaCl_2_	NaHCO_3_	Hepes	Glucose	Protein
Concentrations [mM]	24, 96, 144, 192	24, 96, 144, 192	24, 48, 96	24, 48, 96	24, 96, 144, 192	10, 24, 48, 96	24, 48, 96	10, 50, 100*

*mg/mL.

### Epithelial A6 cells preparation

In the present investigation the effect of aldosterone on the ionic activity was tested on renal epithelial A6 cells derived from Xenopus laevis^21–23^ and purchased from the American Type Culture Collection (ATCC). A6 cells were cultured on plastic flasks at 27 °C in a humidified incubator with 1.0% CO_2_ in air. The culture medium contained 75% (vol/vol) NCTC-109, 15% (vol/vol) distilled water and 10% (vol/vol) fetal bovine serum. Then, we seeded the cells onto culture-treated Transwell-Clear permeable supports (Polycarbonate membrane Transwell-Clear 6.5 and 24 mm), which were obtained from Corning, Inc. (Lowell, MA, USA). The density was quantified as 1 × 10^5^ cells/well. The isotonic test solution contained 120 mMol NaCl, 3.5 mMol KCl, 1 mMol CaCl_2_, 1 mMol MgCl_2_, 5 mMol glucose and 10 mMol N-2-hydroxy-ethylpiperazine-N0 −2-ethanesulfonic acid (HEPES) adjusted to pH 7.4. The dose of aldosterone added to treat the cells was 1 μMol.

### Raman spectroscopic analysis

**Figure 1:**
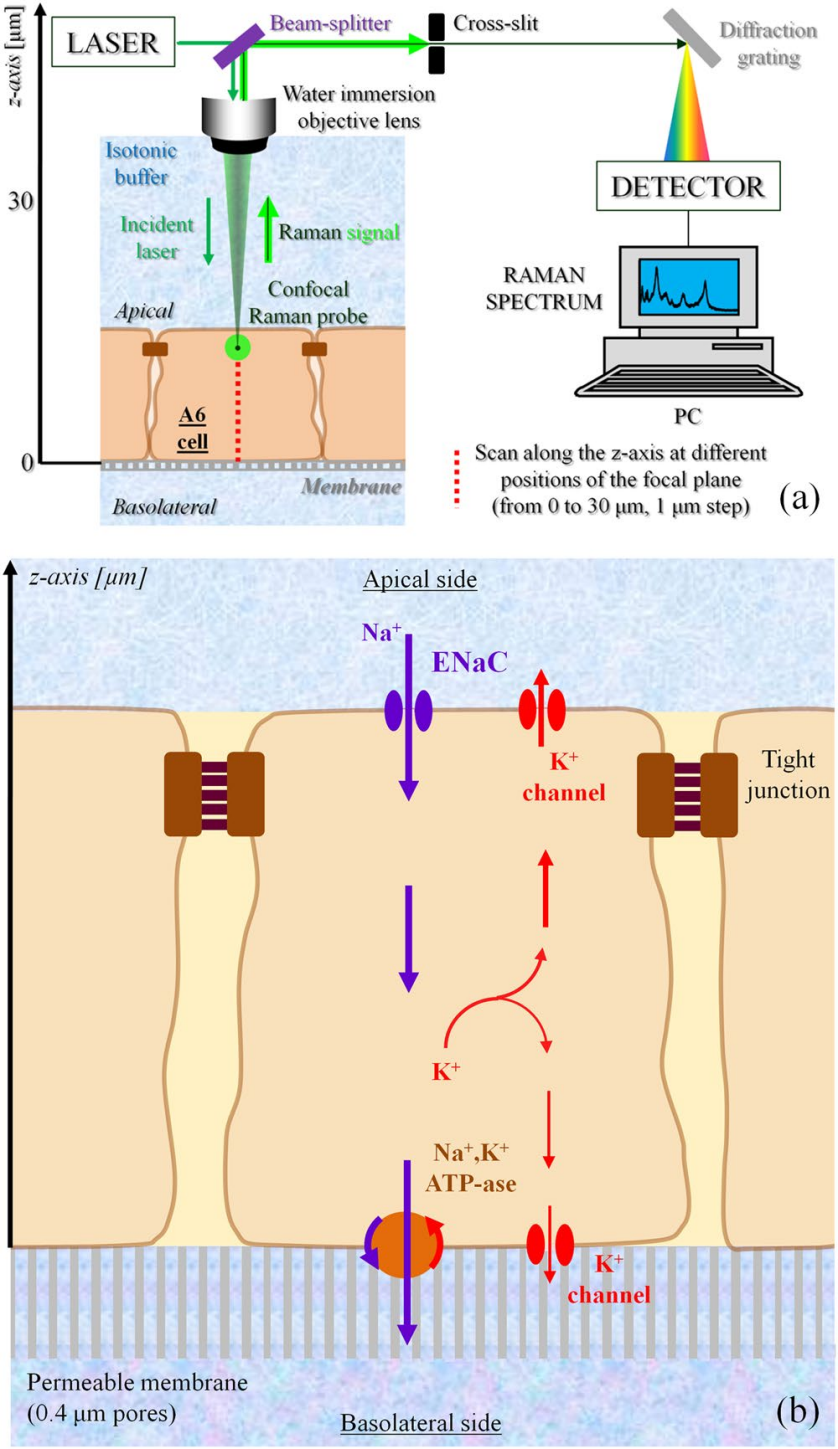
(**a**) Explanation of the z-axis scanning protocol adopted in the Raman spectroscopy experiments. The incident laser and the back-scattered Raman signal collected by the same lens are separated using a beam splitter. The confocal cross-slit enables to increase the spatial resolution of the measurements by cutting off the signal coming from regions that are more than a few hundred of nm out of the focal plane. A diffraction grating is used to split the light into its components, whose intensities are measured by the detector. (**b**) Schematic of the A6 renal epithelial cell under investigation, in which are described the ionic transport mechanism of sodium reabsorption.

**Figure 2:**
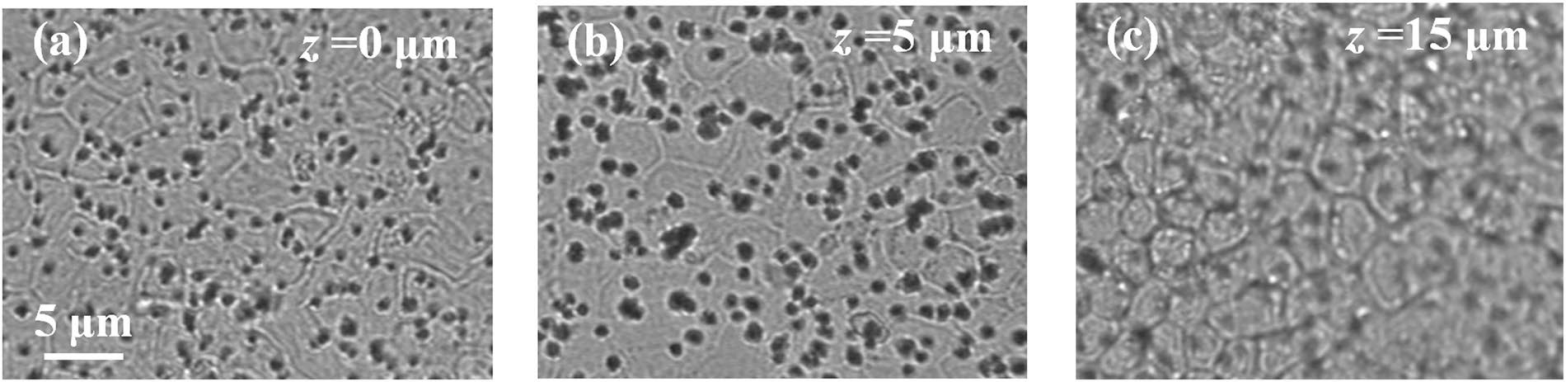
Examples of bright-field images of aldosterone treated cells: (**a**) focus on the permeable membrane (z = 0); (**b**) above the membrane at z = 5 and (**c**) at z = 15 μm.

**Figure 3:**
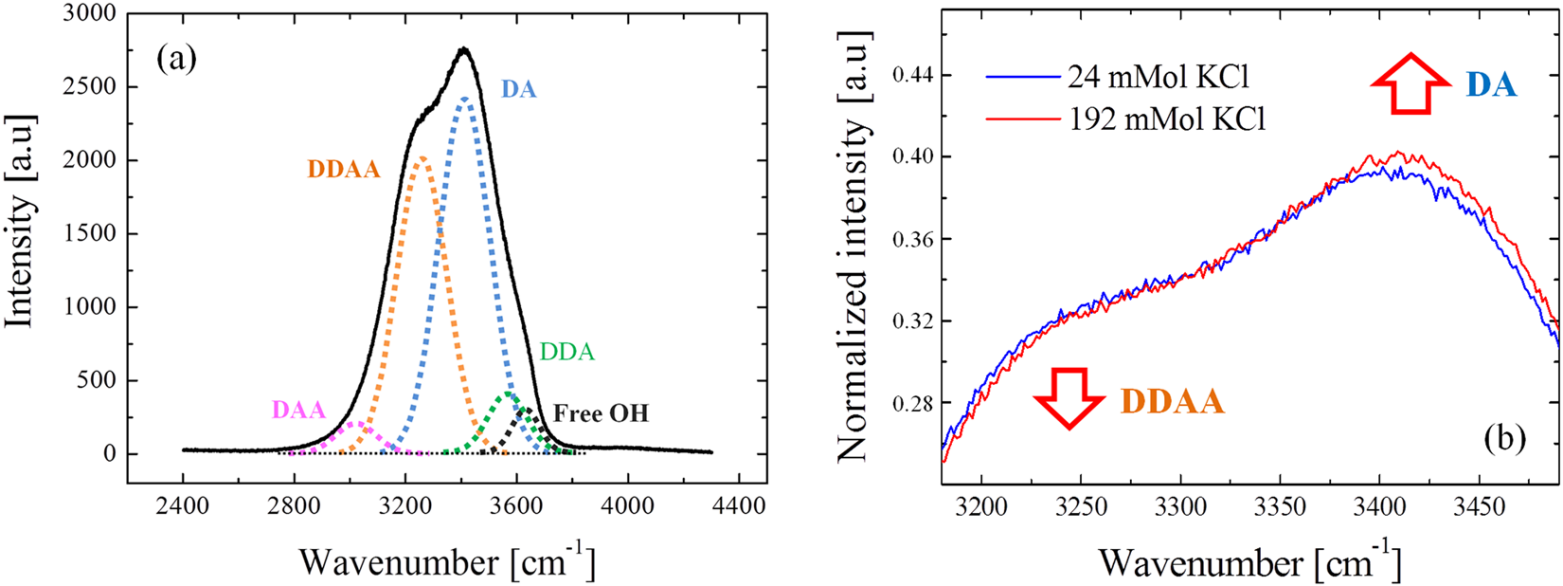
(**a**) Typical Raman spectrum collected from water solutions in the region 2400–4200 cm^−1^ and the 5 sub-bands used to interpret the structure of liquid water. (**b**) Example of spectral variation induced by ionic concentration in the millimolar range (total integrated intensity of each spectrum was normalized to 100).

Raman spectroscopy can be summarily described as the measurement and analysis of the light that is inelastically scattered from molecules illuminated with a suitable monochromatic laser source. The inelastic scattering of the light is correlated to typical vibrations of the molecules probed by the laser. The resulting electromagnetic spectrum collected from the sample is characterized by intensity bands located at different frequencies that depend on the particular kind of vibrations and they can be used as a fingerprint of the molecules. The intensities of the Raman bands depend also on the concentration of the molecules that interact with the incident laser. In the present investigation, Raman spectra were collected using a Raman microscope (Raman-11, Nanophoton, Japan), equipped with a 532 nm laser excitation source. The confocal configuration of the probe adopted throughout the present experiments corresponded to a 60× water immersion objective lens of NA = 1.1 (Olympus, Japan) and cross-slit diameter (Φ) fixed at 70 μm. Before reaching the electrically cooled CCD detector, the scattered light was diffracted utilizing a 600 grooves/mm grating. The in-plane spot size and the axial resolution of the laser probe were estimated using fluorescent beads of 100 nm diameter on a silica glass immersed in deionized water. Briefly, the in-plane laser spot size was estimated by collecting the light emitted during an in-plane raster scan with a step of 100 nm. The measure and plot of the intensity at λ_e_ = 560 nm, which is the emission wavelength of the fluorescent beads, enabled to visualize their positions in the x-y plane. The intensity profile given by a line of points passing through one bead represents the Gaussian shaped laser probe response in the focal plane. The full width at half maximum of the best-fitting Gaussian function can be considered as an estimation of the spot size, which was equal to 0.41 µm. Similarly, the axial resolution of the laser probe was calculated by the intensity profiles collected from z-axis line scans passing through fluorescent beads with a step of 200 nm. In the present work, the Raman spectra were collected with an axial resolution estimated at 1.14 µm. For each solution of the 8 series and the isotonic test buffer, a total of 20 spectra were collected setting the laser power at 70 mW (5 seconds acquisition time for each spectrum). The A6 cells with and without aldosterone were analyzed setting the laser power at approximately 20 mW and collecting spectra from the basal surface up to 30 µm above the permeable membrane (i.e., z-line scans with 1 µm step using a motorized x-y-z stage, 1 second acquisition time). Due to the short acquisition time, we did not cause any damage exposing the cells to the laser source of 20 mW. The two cases of aldosterone treated and untreated cells were studied by comparing the average results obtained from 5 cells for each case. Figure 1(a) shows a schematic of the z-axis scanning protocol adopted to probe the A6 cells. The light scattered from molecules inside the Raman probe was collected by the same objective lens used to focus the incident laser beam. Before reaching the detector, the light passed through the confocal-slit (i.e., pinhole of 70 μm diameter), which enabled to cut-off part of the signal coming from regions outside the laser focal plane, namely improving the axial resolution of the spectrometer. In Fig. 1(b) the ion transport mechanism affected by aldosterone is described in a simplified way to highlight the ions fluxes and the channels involved in the process of Na^+^ reabsorption. Figures 2(a–c) report bright-field images of aldosterone treated cells in which the light was focused on the permeable membrane (i.e., referred as z = 0) and above the membrane at z = 5 and 15 μm. Due to the transparency of the cells, the z-axis coordinate of the plastic permeable substrate in contact with the basolateral membrane was clearly identified by adjusting the focus on the pores (black spots in Fig. 2(a)). Switching from the optical mode to the spectrum acquisition mode, the appearance of the laser spot on the solid plastic surface confirmed the location of the zero coordinate. In the laser focusing procedure, the Airy disk was adjusted with accuracy of 0.1 μm, which is the minimum incremental motion of the stage along the z-axis. Spectral Raman lines were analyzed and deconvoluted using a commercially available software package (Origin 9.1, OriginLab Co., Northampton, MA, USA). Fitting was performed according to Gaussian-Lorentzian (i.e., Voigtian) functions after subtracting a linear baseline. Figure 3(a) shows the typical Raman spectrum collected from water solutions in the region 2400–4200 cm^−1^, which is the spectral range where the strong Raman scattering of the water O-H stretching can be detected. The intricate character of such a broaden band can be inferred by the presence of distinct shoulders, which denotes the convolution of different sub-bands centered at different wavelengths. Since the pioneering studies of Johnston *et al*.^24^ and Woods^25, 26^ during the early 1930s, the O-H stretching vibration of water have been largely investigated and numerous aspects of its complexity have been elucidated. Nonetheless, still we are missing a unanimous interpretation of the sub-bands composing this Raman spectrum. In the present investigation we followed the spectral characterization proposed by Khoshtariya *et al*.^27–29^, which is based on a deconvolution procedure that separates the O-H stretching region into five sub-bands. This interpretation was implemented by Sun^30, 31^, which concluded that the O-H stretching vibration depends on the number and type of hydrogen-bonds in which the water molecule is engaged. A single water molecule can be free of hydrogen bonds or can accept up to a total of 4 hydrogen bonds, which can be named as “donor” (D), if the negatively charged oxygen interacts with the hydrogen of a surrounding molecule, or, conversely, “acceptor” (A), if the positively charged hydrogen binds to the oxygen of a surrounding molecule. The 5 sub-bands showed in Fig. 3(a) are correlated to different clusters of water molecules, each one characterized by a different combination of hydrogen bonds, namely, from lower to higher frequencies, DAA, DDAA, DA, DDA and free OH, respectively. The most favorable structured configurations in liquid water are the DDAA and the DA, located at around 3221 and 3429 cm^−1^, respectively. In the present work, the intensity ratio of the bands DA and DDAA, *r* = *I*_*DA*_ */I*_*DDAA*_, was introduced as a meaningful parameter to investigate the effect of aldosterone on A6 epithelial cells.

### Calculation of the ion flux

In the intracellular fluid, from the apical to the basolateral membrane, the electric potential is constant. Conversely, for a certain ion, chemical potential can be established by concentration gradients, which induces the movement of that ion across the cell. In the present paper, we studied the variations of Na^+^ and Cl^−^ triggered by aldosterone in the cytosol, which were observed by the enhancement of concentration gradients across the cell thickness. The total variations of ionic concentration in the cytosol after treatment by aldosterone, $[{\overline{{\rm{\Delta }}Na}}^{+}]$ and $[{\overline{{\rm{\Delta }}Cl}}^{-}]$, were estimated as follows:

1 $$[{\overline{{\rm{\Delta }}Na}}^{+}]\,{\rm{or}}\,[{\overline{{\rm{\Delta }}Cl}}^{-}]=\frac{{\int }_{0}^{t}{\rm{\Delta }}[N{a}^{+}](z)\,or\,{\rm{\Delta }}[C{l}^{-}](z)dz}{t}$$

where *Δ[Na* ^+^ *](z)* and *Δ[Cl* ^−^ *](z)* are the aldosterone-induced variations of the sodium and chloride concentration profiles from the basolateral to the apical membrane and *t* is the cell thickness in µm. According to the Fick’s first law, the enhancement of Na^+^ flux induced by aldosterone, *ΔJ*_*Na*_, was quantified as follows:

2 $${\rm{\Delta }}{J}_{Na}=-{D}_{Na}\frac{d{\rm{\Delta }}[N{a}^{+}](z)}{dz}$$

where *D*_*Na*_ is the coefficient of diffusion for sodium at 25 °C (i.e., assumed equal to 1.33 × 10^−8^ cm^2^ s^−1^ ^32^) and *dΔ*[*Na* ^+^]*(z)*/*dz* is the concentration gradient stimulated by aldosterone, which was measured by Raman spectroscopy. In A6 epithelial cells treated with aldosterone, *ΔJ*_*Na*_ can be compared to the increase of short-circuit current *ΔI*_*sc*_ measured using the Ussing chamber^33, 34^, by means of the following approximation:

3 $${\rm{\Delta }}{I}_{sc}={\rm{\Delta }}{J}_{Na}\times F$$

where *F* is the Faraday constant equal to 96.485,332 C mol^−1^.

### Statistical analysis

The results of the experiments on the ionic solutions are presented as the mean of n = 20 independent measurements ± standard deviation (SD). In the linear regression analysis, the difference in slopes between two different solutions was statistically validated by calculating the p-value of the interaction term (i.e., ion concentration × type of solution) using the software Minitab^®^17 (Minitab Inc., State College, PA, USA). The data presented for each type of cells (i.e., control and aldosterone treatment) represent the mean of n = 5 independent experiments ± SD. At each z-axis position inside the cytosol, the difference between the mean *r* of the two groups was statistically validated using unpaired *t*-test (p ≤ 0.05).

### Data availability

The datasets generated during and/or analyzed during the current study are available from the corresponding authors on reasonable request.

## Results

**Figure 4:**
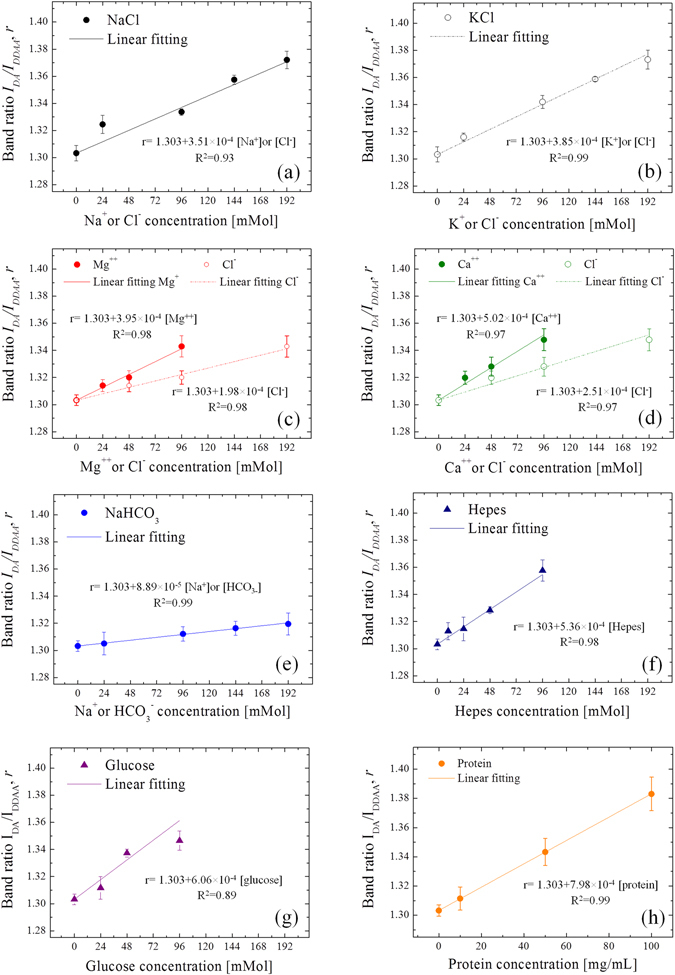
Liner dependence between the ionic concentration and the intensity ratio of the bands DA and DDAA, *r* = *I*_*DA*_ */I*_*DDAA*_, for solutions of NaCl (**a**), KCl (**b**), MgCl_2_ (**c**), CaCl_2_ (**d**), NaHCO_3_ (**e**), Hepes (**f**), glucose (**g**) and proteins (bovine serum albumin) (**h**).

**Figure 5:**
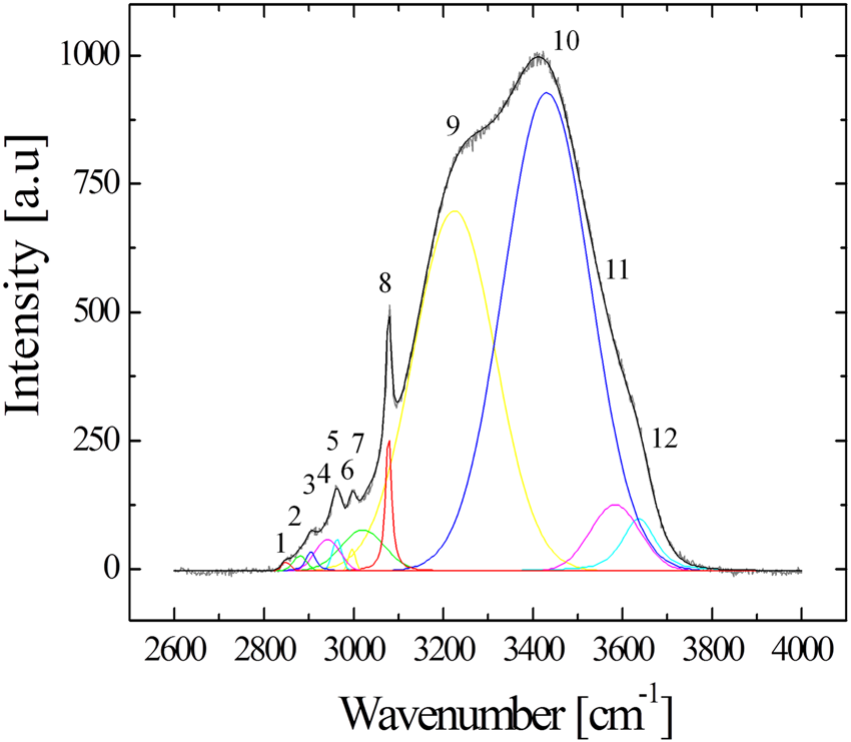
Typical Raman spectrum obtained from A6 cells, focusing the laser probe close to the polycarbonate substrate. Assignments of the labeled bands are reported in Table 2.

**Figure 6:**
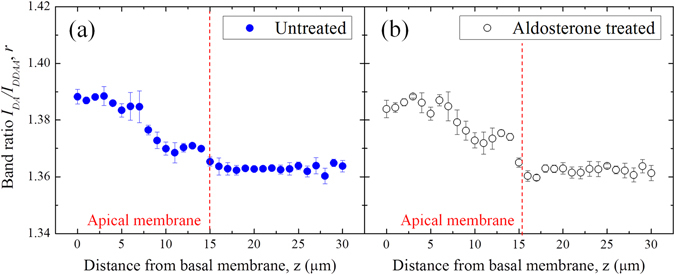
Profiles of the intensity ratio *r*, as retrieved from control (**a**) and aldosterone treated (**b**) A6 cells, respectively.

**Figure 7:**
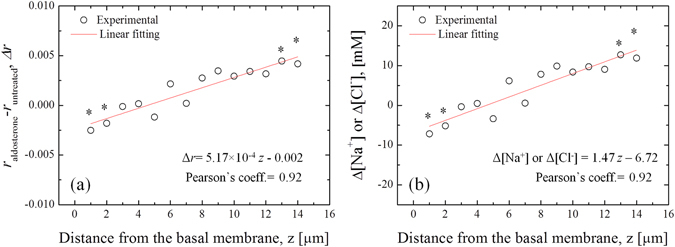
(**a**) Difference of the ratio *r* between treated and untreated cells as calculated in the cytosol of A6 epithelial cells. (**b**) The data reported in (**a**) can be converted to variation of [Na^+^] or [Cl^−^] in the cytosol using the linear fitting equation reported in the inset of Fig. 3(a). At each z-axis coordinate, the statistically meaningful variations of *r* and [Na] (or [Cl^−^]) are marked by *(unpaired t-test, 95% confidence, p < 0.05).

In previous studies it was clearly demonstrated that the intensities of the O-H stretching bands are affected by the presence of ions in solutions, which alters the populations of the water clusters^30, 35, 36^. Figure 3(b) reports an example of variation of the Raman spectra for solutions containing 24 and 192 mMol of KCl. In the present investigation, the Raman spectroscopic experiments on different ionic solutions in the millimolar range show linear dependence between the ionic concentration and the intensity ratio *r*, as can be seen in Fig. 4(a–h) for the series 1–8, respectively. The linear regression analysis show that NaCl and KCl have statistically equal slopes (p-value of the interaction term >0.05). The ratio *r* for MgCl_2_ and CaCl_2_ solutions was plotted as a function of [Cl^−^], [Mg^+^] and [K^+^], in order to compare data with equal cation or anion concentrations, but obtained from different solutions. Such a comparison was aimed at clarifying which type of ion had the major contribute to the variation of *r*. The difference between the slopes of MgCl_2_ and CaCl_2_ solutions is not statistically meaningful (p > 0.05). In addition, the slopes are equal to those of NaCl and KCl when *r* is plotted as a function of cation concentration, although the Cl^−^ concentration is as half as much. Conversely, the slope of the fitting line for NaHCO_3_ is statistically the smallest among those of the investigated solutions. Hepes and glucose show statistically stronger effect on the variation of *r* as compared to the other ion solutions at equal mMol. The solutes taken into consideration for the calibrations of the parameter *r*, with the exception of proteins, were used to prepare the isotonic buffer for testing the A6 cells, whose composition was previously reported in the paragraph *Epithelial A6 cells preparation*. Using these concentrations and the linear equations showed in Fig. 4, the sum of the intensity ratios *r* of each component was calculated as 1.362, while the experimental value obtained from the buffer solution is 1.363. As already explained in the paragraph *Raman spectroscopic analysis*, the experiments with the A6 epithelial cells consisted in the acquisition of Raman spectra from the basolateral membrane up to the extracellalur fluid over the apical membrane. In the high frequencies range considered for this study, the C-H stretching vibrations of lipids, proteins and the polycarbonate membrane overlap the water O-H stretching Raman spectrum in the range 2800–3200 cm^−1^, as shown in Fig. 5, which reports a typical spectrum obtained focusing the laser probe close to the polycarbonate substrate. The fitting procedure enabled us to separate the contributions of each band from the total spectral line, according to the vibrational assignments reported in Table 2 (see also the sub-bands labeled in Fig. 5)^37–41^. In Fig. 6(a,b), the average profiles of band intensity ratio *r* along the z-axis were plotted for aldosterone untreated and treated cells, respectively. In both cases, *r* reaches a constant value for *z* > 15 µm, which is consistent to the ratio calculated from the isotonic test solution, namely 1.363. Such experimental evidence was used to estimate the position of the apical membrane, which is approximated to 15 µm in aldosterone treated and untreated cells. According to these considerations, the most striking evidence is the evolution of this ratio inside the cell (i.e., *z* < 15 µm), which has two distinct patterns depending on the presence of aldosterone. In the case of untreated cells, the highest values of *r* were detected close to the basolateral membrane, while in the upper intracellular fluid the ratio is slightly higher than the average retrieved from the extracellular space. Conversely, the addition of aldosterone indisputably increased the *r* parameter in the proximity of the apical membrane. In Fig. 7(a) the effect of aldosterone is clearly highlighted by the difference of *r* between treated and untreated cells (hereafter referred as $\Delta r$) as a function of the distance from the basolateral membrane. The intensities of the CH_2_ and CH_3_ symmetric stretching bands of proteins and lipids located at 2855 and 2874 cm^−1^ did not denote any detectable increase in the proximity of the apical surface of the treated cells, namely the increase of proteins induced by aldosterone was not sufficiently large to be detected using confocal Raman spectroscopy. Based on this consideration, we hypothesized that the perturbation of the water Raman spectrum observed after the addition of aldosterone might be merely ascribable to the activity of sodium and chloride in the cytosol of the epithelial cells and we considered the contribution from the increase of proteins as negligible. Since the electroneutrality condition has to be maintained, we assumed that, at each depth of the cytosol, the local variation of Na^+^ induced by aldosterone was balanced by an equal amount of Cl^−^. Accordingly, Fig. 7(b) reports the variation of sodium concentration induced by aldosterone along the cell thickness as calculated using $\Delta r$ and the equation reported in Fig. 4(a) for NaCl. As compared to the untreated cells, the addition of aldosterone promoted a linear variation of [Cl^−^] and [Na^+^] from the apical to the basolateral membrane (Pearson’s linear correlation coefficient = 0.92), with concentration increasing toward the apical side of the cell and ionic depletion toward the basolateral side. As a result, the average variations of sodium and chloride in the cytosol, $[{\overline{{\rm{\Delta }}\mathrm{Na}}}^{+}]$ and $[{\overline{{\rm{\Delta }}\mathrm{Cl}}}^{-}]$, were estimated integrating the linear fitting equation of Fig. 7(b) as explained in Eq. 1. The result is an increase of chloride and sodium of 4.3 mMol. The enhancement of Na^+^ flux induced by aldosterone, *ΔJ*_*Na*_, was quantified using Eq. 2, in which the concentration gradient stimulated by aldosterone, *dΔ*[*Na* ^*+*^]*(z)*/*dz*, was −1.47 mMol µm^−1^ (see the slope in Fig. 7(b)). Note that the gradient is negative in the calculation of the flux, since by convention the *z-axis* is positive from the apical to the basolateral surface. Based on the Raman microscopic assessments, *ΔJ*_*Na*_ was quantified at 1.95 × 10^−10^ mol s^−1^ cm^−2^. In addition, using *ΔJ*_*Na*_ in Eq. 3 we estimated the enhancement of short-circuit current, *ΔI*_*sc*_, at 18.8 µA cm^−2^.

**Table 2:** Main Raman bands observed in the spectra collected during the A6 cells analysis and their assignments (bands numbered according to Fig. 5).

***Band label***	***Wavenumber [cm*** ^***−1***^ ***]***	***Assignment***
1	2855	CH_2_ ν_s_ − L, P^34, 35^
2	2874	CH_3_ ν_s_ and R_3_C-H, −L, P^34, 35^
3	2900	CH_2_ ν_as_ − L, P and CH_3_ ν_as_ − PC^34, 37^
4	2933	CH_3_ ν_as_ − L and P^34, 35^
5	2980	Strong aromatic C-H stretch, PC^36–38^
6	3000	Strong aromatic C-H stretch, PC^37, 38^
7	3016	O-H stretching, H_2_O, DAA^30, 31^
8	3072	Strong aromatic C-H stretch, PC^37, 38^
9	3221	O-H stretching, H_2_O, DDAA^30, 31^
10	3429	O-H stretching, H_2_O, DA^30, 31^
11	3572	O-H stretching, H_2_O, DDA^30, 31^
12	3636	O-H stretching, H_2_O, free O-H^30, 31^

P = proteins, L = lipids, PC = polycarbonate, ν_s_ = symmetric stretching, ν_as_ = asymmetric stretching.

## Discussion

The present spectroscopic investigation introduced for the first time the use of the O-H stretching vibration of water to extrapolate meaningful information concerning the effect of aldosterone on epithelial cells. Since monitoring the ionic transport and activity that occur in the microscopic intracellular space was the matter of interest in this study, Raman spectroscopy offered the advantage of having high spatial resolution and being a nondestructive technique, capable of probing the cells through their thickness in a nimble way. The drawback of this technique resides in the fact that it cannot directly detect the presence of monovalent ions, since they are not molecules, namely they do not generate molecular vibrations. As previously mentioned, although we are still lacking of a unanimous interpretation of the O-H stretching of water in all its facets, it has been proved that the presence of ions clearly alters the vibrations of the water molecules, which represent the solvent in which ionic species are dissolved. The complete rationalization of the data presented in the previous section necessitates the elucidation of some basic aspects of the interaction between solvent and solute, and, in particular, the general concept of ions “structure maker” or “structure breaker”^42–50^. The structure maker ions favor the water network that resembles the structure of ice, where every water molecule is connected by four H-bonds to its neighbors in a tetrahedral arrangement, which is the configuration related to the DDAA Raman band. Conversely, the structure breaker ions can disrupt hydrogen bonds also beyond the first hydration shell, increasing the population of molecules with less than four hydrogen bonds, which is mostly represented by the DA Raman band^51^. As a general rule, the most cations are structure makers, with the exception of some alkali metals. In fact, while sodium is slightly structure maker, potassium is slightly structure breaker^47, 48^. Anions with high charge density are structure makers, while monovalent anions, like Cl^−^, are structure breakers, with the exceptions of fluoride and hydroxide. Based on these considerations, the salient features obtained from the linear calibrations of the solutions shown in Fig. 4(a–h), can be summarized as follows:

i. At very low concentrations, the difference between Na^+^ and K^+^ was not detected in terms of *r* variation, although the former is slightly structure maker and the latter is slightly structure breaker.
ii. Since the linear correlations in Fig. 4(a,b) are similar, water structure breaker Cl^−^ is overriding the effect of Na^+^ and K^+^.
iii. Conversely, Mg^++^ and Ca^++^ have a detectable structure maker behavior: in fact, compared to Na^+^ and K^+^ solutions with equal Cl^−^ concentration, *r* is clearly lower (see the dash-dot lines in Fig. 4(c,d)).
iv. HCO_3_^−^, as a monovalent anion, is structure breaker, but less dominant than Cl^−^ (see Fig. 4(e)).
v. Proteins, Glucose and Hepes are both strongly structure breakers (see Fig. 4(f,g,h)), as expected considering the presence of strong electronegative hydroxyls^50^.

With these notions in mind, we can explain the data obtained from the analyses of the A6 cells by considering the ratio *r* as an “ionic activity index”. In fact, the only ions that may decrease *r* with their increasing concentration are Ca^++^ and Mg^++^, but their contribution to the total activity is negligible due to their minimal expected concentrations in the intracellular and extracellular fluids. The study of bovine serum albumin in solution was specifically conceived to verify that the major increase of *r* in the cytosol as compared to the extracellular fluid can be explained by the strong contribute of proteins to the perturbation of the H_2_O Raman spectrum. As already mentioned, any possible increase of proteins concentration due to aldosterone was not sufficient to effectively alter the Raman spectrum. In Fig. 6(b), as compared to the results of the untreated cells showed in Fig. 6(a), the higher values of the ratio *r* detected near the apical surface of the aldosterone treated cells may be correlated to the up-regulation of ENaC, which led to the increase of Na^+^ uptake and release of K^+^, since the levels of potassium channels in the apical membrane (i.e., ROMK channels) are not inhibited due to the absence of angiotensin II^12^. The enhanced flux of Cl^−^ in the apical membrane also explains the increase of *r*, which is greatly affected by this anion. In the present study, as reported in the section *Results*, we correlated the variation of *r* between treated and untreated cells to the variation of sodium and chloride space distribution in the cytosol induced by aldosterone. Such a correlation enabled us to visualize the effect of this hormone on the ionic transepithelial transport, which can be summarily explained by the concomitant activation and up-regulation of apical ENaCs and basolateral Na^+^/K^+^ ATP-ase (pumps), which enhanced Na^+^ absorption in the apical side and Na^+^ excretion in the basolateral membrane, respectively. The overall increase of the ionic concentration gradient detected from the apical to the basolateral sides reflects the ionic flux across the intracellular space, since we can consider the electric potential inside the cell as constant and the chemical potential of each ion as the driving force of the flux across the epithelial cell. The standard methods to calculate the ionic flux in epithelial cells relies on electrophysiology and liquid scintillation spectrometry. In A6 epithelial cells, the difference of *I*_*sc*_ measured between aldosterone treated and untreated cells, Δ*I*_*sc*_, can be associated to the net Na^+^ flux across the cell, from the lumen to the interstitial space. In other words, the flux and the current estimated using Eqs [2 and 3] can be compared to those obtained by conventional electrophysiological measurements. Devuyst *et al*.^34^ investigated the effects of aldosterone (0.1 µMol) on sodium transport and chloride permeability in A6 cells measuring the transepithelial potential difference and the short-circuit current. The difference of *I*_*sc*_ between treated and untreated cells was 7.9 µA cm^−2^. Similarly, Bindels *et. al*.^33^ measured an increase of *I*_*sc*_ equal to 12 µA cm^−2^. Conversely, De La Rosa *et al*.^52^ reported an increase of 31.3 µA cm^−2^, which is higher than 18.8 µA cm^−2^ estimated in the present study. Overall, all the values obtained by different methods from different cultures of A6 cells showed a comparable enhancement of Na^+^ absorption, which demonstrates that Raman spectroscopy is a viable non-destructive tool to investigate the effect of aldosterone on the ionic activity in the intracellular space. In addition, according to the results showed in Fig. 6, Raman spectroscopy showed that the treatment with aldosterone did not alter the cell thickness, confirming the data reported by Schneider *et al*.^53^, which detected volume increase in endothelial cells by the atomic force microscope only during the first 25 minutes of aldosterone exposure. Our Raman spectroscopic analysis was performed 4 hours after the addition of the hormone. Further developments of this method are required to elucidate the dynamics of the effect of aldosterone during the first minutes or after a long period of treatment. In the present paper the effect of aldosterone was addressed as a preliminary example of the use of Raman spectroscopy to study epithelial cells. The potential of this non-destructive method of analysis can be easily exploited to deeply investigate the effects of different treatments, such as the effect of vasopressin on the ENaC surface expression in A6 cells^17^.

## Conclusions

The present investigation established new methods based on Raman microspectroscopy to monitor the transcellular ion transport in A6 epithelial renal cells treated with aldosterone. The sensitivity of the vibrational behavior of water to the variation of ions in the millimolar range was used to detect the increase of ionic activity in the intracellular fluid, which can be stimulated by different treatment aimed at investigating the regulation of ion channels and ion pumps. Based on the preliminary Raman spectroscopic investigations of physiologically relevant solutions containing different ionic species, the variation of the water Raman spectrum detected at different locations of the cytosol have been correlated to the perturbation of sodium and chloride concentrations induced by aldosterone. The enhanced Na^+^ flux was estimated from gradients of chemical potential measured inside the cells and it was converted to current density comparable to the short-circuit current that can be measured using electrophysiological methods. The results obtained using the Raman spectroscopic technique are similar to the typical data reported in literature.
